# Lysine Acetyltransferase GCN5b Interacts with AP2 Factors and Is Required for *Toxoplasma gondii* Proliferation

**DOI:** 10.1371/journal.ppat.1003830

**Published:** 2014-01-02

**Authors:** Jiachen Wang, Stacy E. Dixon, Li-Min Ting, Ting-Kai Liu, Victoria Jeffers, Matthew M. Croken, Myrasol Calloway, Dominique Cannella, Mohamed Ali Hakimi, Kami Kim, William J. Sullivan

**Affiliations:** 1 Department of Pharmacology & Toxicology, Indiana University School of Medicine, Indianapolis, Indiana, United States of America; 2 Department of Medicine, Albert Einstein College of Medicine, Bronx, New York, New York, United States of America; 3 Department of Microbiology & Immunology, Albert Einstein College of Medicine, Bronx, New York, New York, United States of America; 4 Laboratory for Macromolecular Analysis, Albert Einstein College of Medicine, Bronx, New York, New York, United States of America; 5 Université Joseph Fourier, Grenoble, France; 6 Microbiology & Immunology, Indiana University School of Medicine, Indianapolis, Indiana, United States of America; Pasteur Institute Lille, France

## Abstract

Histone acetylation has been linked to developmental changes in gene expression and is a validated drug target of apicomplexan parasites, but little is known about the roles of individual histone modifying enzymes and how they are recruited to target genes. The protozoan parasite *Toxoplasma gondii* (phylum Apicomplexa) is unusual among invertebrates in possessing two GCN5-family lysine acetyltransferases (KATs). While GCN5a is required for gene expression in response to alkaline stress, this KAT is dispensable for parasite proliferation in normal culture conditions. In contrast, GCN5b cannot be disrupted, suggesting it is essential for *Toxoplasma* viability. To further explore the function of GCN5b, we generated clonal parasites expressing an inducible HA-tagged dominant-negative form of GCN5b containing a point mutation that ablates enzymatic activity (E703G). Stabilization of this dominant-negative GCN5b was mediated through ligand-binding to a destabilization domain (dd) fused to the protein. Induced accumulation of the _ddHA_GCN5b(E703G) protein led to a rapid arrest in parasite replication. Growth arrest was accompanied by a decrease in histone H3 acetylation at specific lysine residues as well as reduced expression of GCN5b target genes in GCN5b(E703G) parasites, which were identified using chromatin immunoprecipitation coupled with microarray hybridization (ChIP-chip). Proteomics studies revealed that GCN5b interacts with AP2-domain proteins, apicomplexan plant-like transcription factors, as well as a “core complex” that includes the co-activator ADA2-A, TFIID subunits, LEO1 polymerase-associated factor (Paf1) subunit, and RRM proteins. The dominant-negative phenotype of _ddHA_GCN5b(E703G) parasites, considered with the proteomics and ChIP-chip data, indicate that GCN5b plays a central role in transcriptional and chromatin remodeling complexes. We conclude that GCN5b has a non-redundant and indispensable role in regulating gene expression required during the *Toxoplasma* lytic cycle.

## Author Summary

*Toxoplasma gondii* is a protozoan parasite that causes significant opportunistic infection in AIDS and other immunocompromised patients. Acute episodes of toxoplasmosis stem from tissue destruction caused by the rapidly growing form of the parasite, the tachyzoite. In this study, we identify a lysine acetyltransferase (KAT) enzyme called GCN5b that is an essential driver of tachyzoite proliferation. Our studies show that GCN5b is present at a wide variety of parasite genes and that expression of defective GCN5b compromises gene expression through its diminished ability to acetylate histone proteins. We also identified the likely mechanism by which GCN5b is recruited to target genes by co-purifying this KAT with plant-like AP2-domain proteins, a subset of which function as DNA-binding transcription factors in Apicomplexa. Our findings demonstrate that KATs play a critical role in parasite replication, which leads to tissue destruction and acute disease in the host. Parasite KAT enzyme complexes may therefore serve as attractive targets for future drug development.

## Introduction

Lysine acetylation of histones is a well-characterized post-translational modification linked to the activation of gene expression. Initially identified in the free-living protozoan *Tetrahymena thermophila*, the first histone acetyltransferase (HAT) was homologous to a transcriptional adaptor protein in yeast known as GCN5 [1]. It has since been elucidated that GCN5 is a highly conserved catalytic component present in multiple protein complexes linked to the regulation of gene expression [2]. When GCN5 HATs were found to have non-histone substrates as well, they became referred to as lysine (K) acetyltransferases (KATs) [3]. The number of GCN5 KATs and their impact on cells or organisms depends on the species. GCN5 generally appears to be required for stress responses [4]–[6]. Consistent with this idea, the single GCN5 is dispensable in *Saccharomyces cerevisiae*, but required for growth on minimal media [7]. In contrast, mammals possess two GCN5 family members, one of which is required for mouse embryogenesis [8]. Null mutants of the other GCN5 (also known as PCAF, or p300/CBP-associating factor) have no discernible phenotype in mice [8], [9]. Together, these reports suggest that GCN5-mediated acetylation is an important facet of cellular biology, particularly during stress or adaptive responses.

The relevance of lysine acetylation in pathogenic protozoa is underscored by potent antiprotozoal activity of lysine deacetylation inhibitors like apicidin and FR235222 [10], [11]. Histone acetylation has also been linked to a number of processes that underlie pathogenesis of apicomplexan parasites, including antigenic variation in *Plasmodium* (malaria) and developmental transitions in *Toxoplasma gondii* [5], [12], [13]. An extensive repertoire of histone modification machinery is present in these parasites, suggesting that epigenetic-based regulation contributes to gene expression control [14]. A related oddity of the Apicomplexa is that these early-branching eukaryotes appear to use an expanded lineage of so-called Apetela-2 (AP2) proteins as transcription factors rather than the basic leucine zipper (bZIP) factors that are conserved throughout most of the eukaryotic kingdom [15], [16]. ApiAP2 proteins harbor a plant-like DNA-binding domain and emerging evidence supports that at least some function as *bona fide* transcriptional regulators [17], [18].

*Toxoplasma* has a number of unusual features with respect to its GCN5 KATs. First, there are two GCN5-family members in *Toxoplasma* (GCN5a and b) whereas other invertebrates, including *Plasmodium*, possess only one [19], [20]. Second, both TgGCN5s have long N-terminal extensions devoid of known protein domains. These N-terminal extensions are not homologous to those seen in higher eukaryotes, nor are they homologous to each other or other apicomplexan GCN5s [20]. One function of the TgGCN5 N-terminal extensions is to translocate the KAT into the parasite nucleus via a basic-rich nuclear localization signal [21], [22]. Yeast two-hybrid studies have suggested that the N-terminal extension of *Plasmodium* GCN5 plays a major role in mediating protein-protein interactions [23]. We previously generated a gene knockout of GCN5a, but similar methods have not produced viable GCN5b knockouts. GCN5a was found to be dispensable for parasite proliferation *in vitro*, but required for the parasite to respond properly to alkaline stress [5]. These findings are consistent with the well-documented role of GCN5 KATs in the cellular stress response.

The inability to knockout GCN5b suggested it is essential for parasite viability. To gain a better understanding of its function in parasite physiology, we expressed a dominant-negative form of GCN5b that lowered histone acetylation, altered gene expression, and arrested parasite proliferation. We also biochemically purified the multi-subunit GCN5b complex to identify interacting proteins and performed a genome-wide ChIP-chip analysis. Collectively, these findings establish that the KAT GCN5b interacts with AP2 factors to regulate the expression of a wide variety of genes that are essential for parasite replication.

## Results

### Expression of catalytically inactive GCN5b arrests replication

To define the role of GCN5b in *Toxoplasma*, we attempted to generate a gene knockout. Repeated attempts to disrupt or replace GCN5b using homologous recombination in haploid type I RH strain tachyzoites have not been successful, in contrast to our ability to knockout GCN5a using the same approach [5]. More recent attempts to knockout the GCN5b locus in a Δ*ku80* background also failed to generate viable parasites, further suggesting that GCN5b is essential in tachyzoites.

We then pursued an inducible dominant-negative strategy to ascertain the importance of GCN5b in *Toxoplasma*. As GCN5 KATs function in multi-subunit complexes, GCN5b is a good candidate for a dominant-negative strategy whereby an ectopically expressed enzymatically dead version would compete for essential interacting proteins from the endogenous protein. Consequently, the activities of the endogenous GCN5b complex would be attenuated. We generated clonal parasites in an RH background expressing a catalytically inactive form of GCN5b (mutated glutamic acid 703 to glycine, E703G [24]) fused to a destabilization domain and HA tag (ddHA) at the N-terminus. *In vitro* HAT assays using purified _ddHA_GCN5b proteins confirm the E703G mutation ablates enzymatic activity (Supplemental Figure S1). We also generated a clone expressing wild-type (WT) GCN5b in the same fashion to serve as a control in phenotypic analyses.

**Figure 1:**
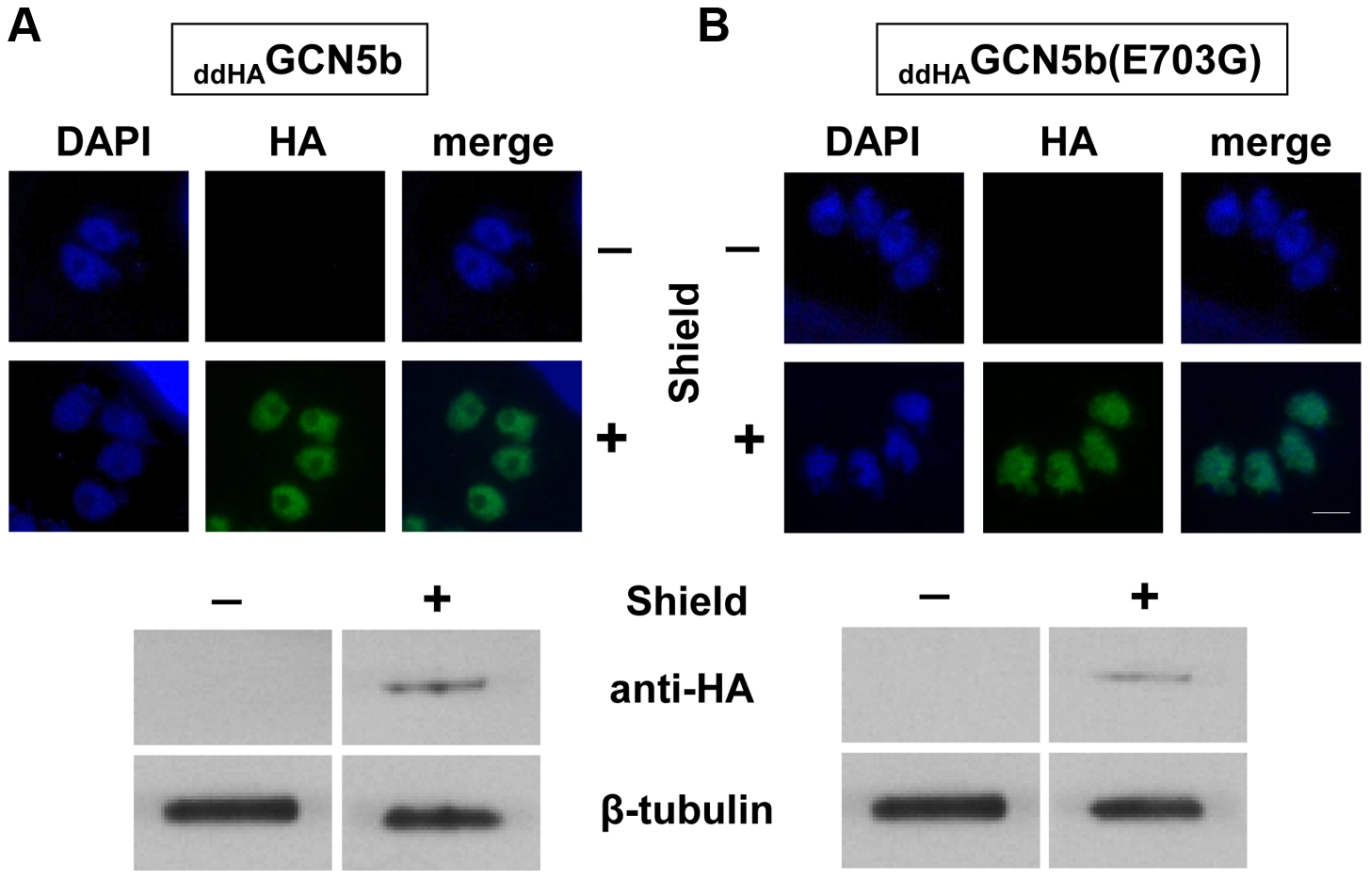
Inducible expression of ectopic _ddHA_GCN5b and _ddHA_GCN5b(E703G) in *Toxoplasma*. Immunofluorescence assays (IFAs) and Western blots using an anti-HA antibody on (A) _ddHA_GCN5b and (B) _ddHA_GCN5b(E703G) parasites, cultured in the presence (+) or absence (−) of 500 nM Shield for 48 hours. Anti-HA signal is shown in green and nucleic acid staining with DAPI is depicted in blue for the IFAs. Scale bar = 2 µm. Antibodies against β–tubulin were used as a loading control on the Western blots.

**Figure 2:**
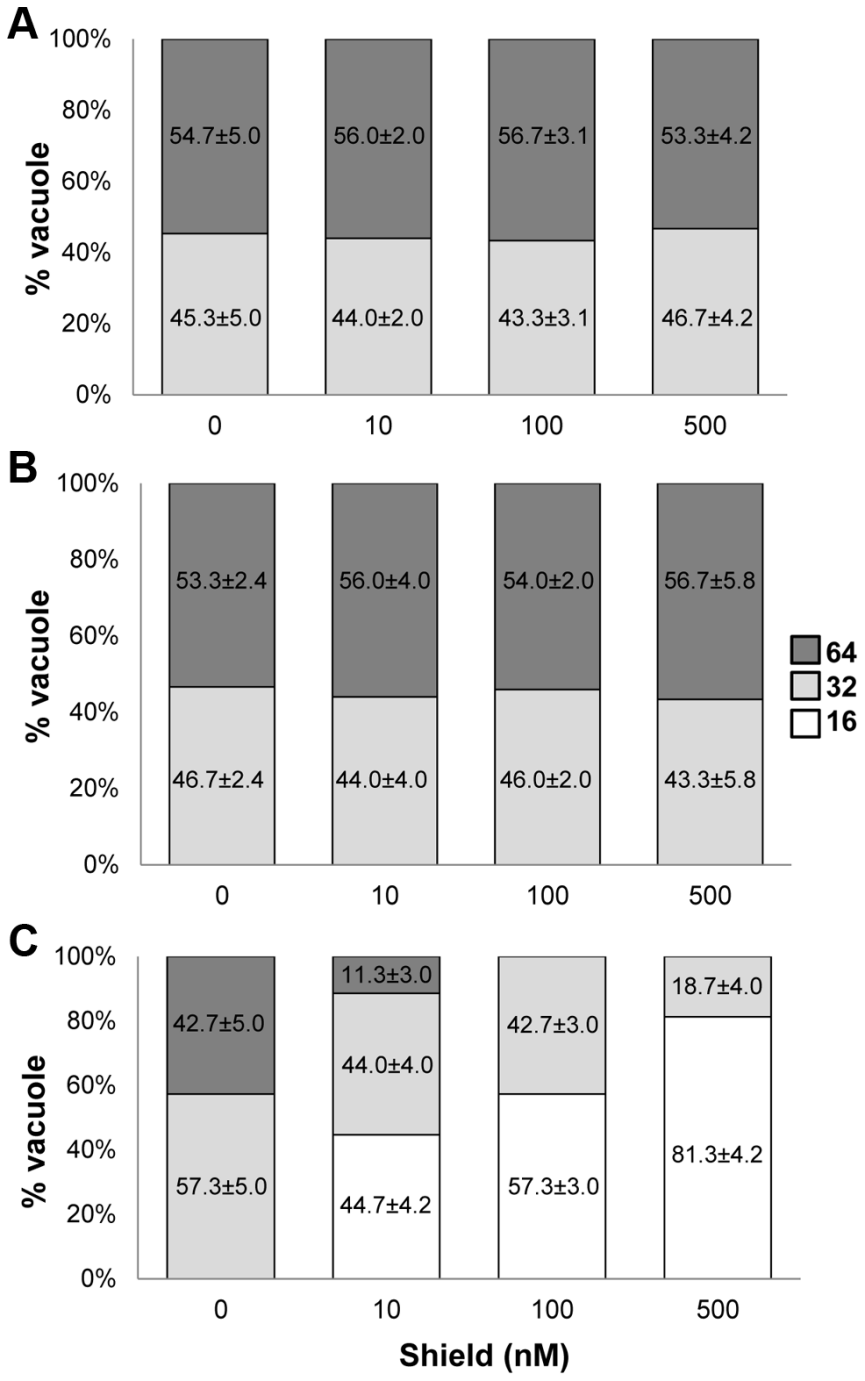
Induced expression of _ddHA_GCN5b(E703G) arrests parasite replication. Doubling assays were performed to assess growth of (A) parental wild-type, (B) _ddHA_GCN5b, and (C) _ddHA_GCN5b(E703G) parasites. Intracellular parasites were physically released from host cells and an equal number of parasites were allowed to infect fresh HFF monolayers. The infected cultures were subjected to the indicated Shield concentrations for 48 hr. Parasite proliferation was monitored by quantifying the numbers of parasites in 50 random vacuoles.

The dd domain directs its fusion partner to the proteasome for rapid degradation, but this can be averted by adding Shield ligand to the culture medium [25]. Fusion of ddHA to the N-terminus of GCN5b or GCN5b(E703G) allowed their ectopic expression to be regulated via Shield, as assessed in immunofluorescence assays (IFAs) and immunoblots using anti-HA (Fig. 1). Fusion of ddHA did not disrupt nuclear localization of WT or mutant GCN5b (Fig. 1). No difference in parasite replication was observed between parental wild-type parasites and those expressing ectopic _ddHA_GCN5b protein at any concentration of Shield (Fig. 2A and 2B). In contrast, parasites induced to express _ddHA_GCN5b(E703G) underwent rapid growth arrest in 48 hours with as little as 10 nM Shield (Fig. 2C). At 500 nM Shield, over 80% of the parasite vacuoles contained only 16 parasites, compared to the control in which most vacuoles contained 64 parasites. Similar results were obtained when we used a PCR-based assay for the B1 gene to measure parasite replication [5] (data not shown). The growth arrest observed for the Shield-treated _ddHA_GCN5b(E703G) parasites is reversible, as parasite plaques were evident in monolayers 48 hours after removal of Shield (Supplemental Figure S2). No plaques were present in monolayers infected with _ddHA_GCN5b(E703G) parasites that were maintained on Shield. As an additional control, _ddHA_GCN5b(E703G) parasites were treated with 1.0 µM pyrimethamine for 48 hours, which irreversibly kills the parasites as indicated by no plaque formation after removal of the drug (Supplemental Figure S2). These studies indicate that induction of the dominant-negative GCN5b attenuates the activity of the endogenous GCN5b complex, which results in stalled replication. However, as suggested by the genetic studies, complete ablation of GCN5b is not tolerated by tachyzoites.

### Reduced histone acetylation in GCN5b dominant-negative parasites

**Figure 3:**
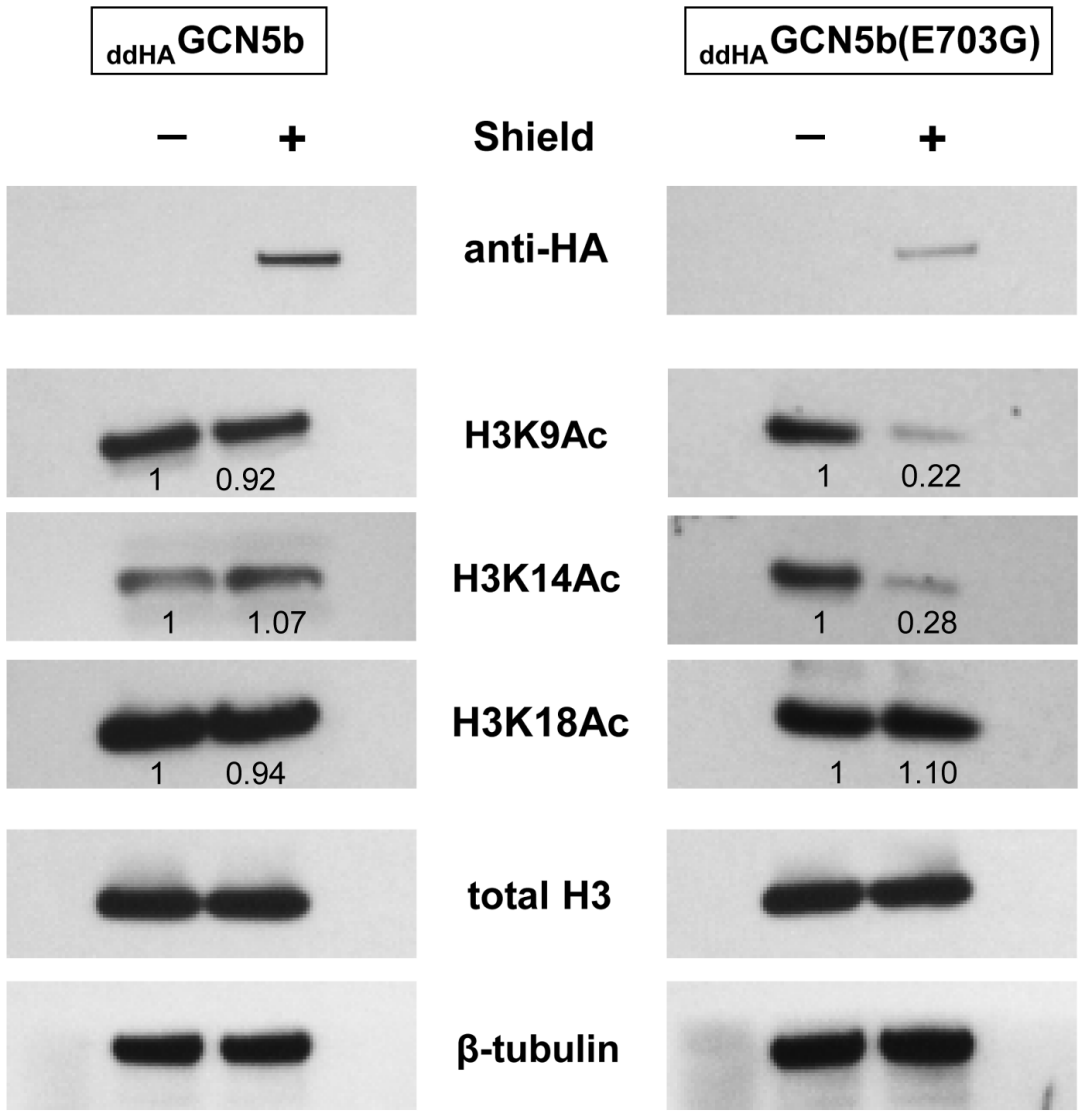
Reduced histone H3 acetylation in _ddHA_GCN5b(E703G) parasites. _ddHA_GCN5b and _ddHA_GCN5b(E703G) parasites were cultured in the presence of 500 nM Shield for 48 hr. Equivalent amounts of parasite lysate obtained from Shield- vs. vehicle-treated parasites were analyzed by Western blotting using antibodies recognizing various acetylated lysine (K) residues of histone H3 (acetylated K9, K14, and K18). Anti-HA was used to monitor protein stabilization. Antibodies to total H3 show that induced stabilization of each GCN5b protein did not alter overall H3 protein levels. Western analysis of β-tubulin levels served as an additional loading control. Densitometry was used to compare relative levels of each histone acetylation mark between the Shield- and vehicle-treated parasites normalized to β-tubulin.

We considered that the replication arrest observed in _ddHA_GCN5b(E703G) parasites may be due to a reduction in histone acetylation, which in turn would lead to dysregulation of the transcriptome. To assess this possibility, we analyzed the acetylation level of individual lysine residues in histone H3, the preferred substrate of GCN5 family KATs, purified from Shield- versus vehicle-treated _ddHA_GCN5b or _ddHA_GCN5b(E703G) expressing parasites. While total levels of H3 protein remain unaltered, acetylation of lysines K9 and K14 was specifically reduced in parasites expressing _ddHA_GCN5b(E703G) (Fig. 3). Interestingly, the acetylation status of H3K18 was not affected, which may be explained by the fact that GCN5a, which would not be attenuated by the expression of _ddHA_GCN5b(E703G), has an exquisite affinity for this particular lysine residue on H3 [20]. These data indicate that the expression of _ddHA_GCN5b(E703G) protein diminishes acetylation on histone H3.

**Figure 4:**
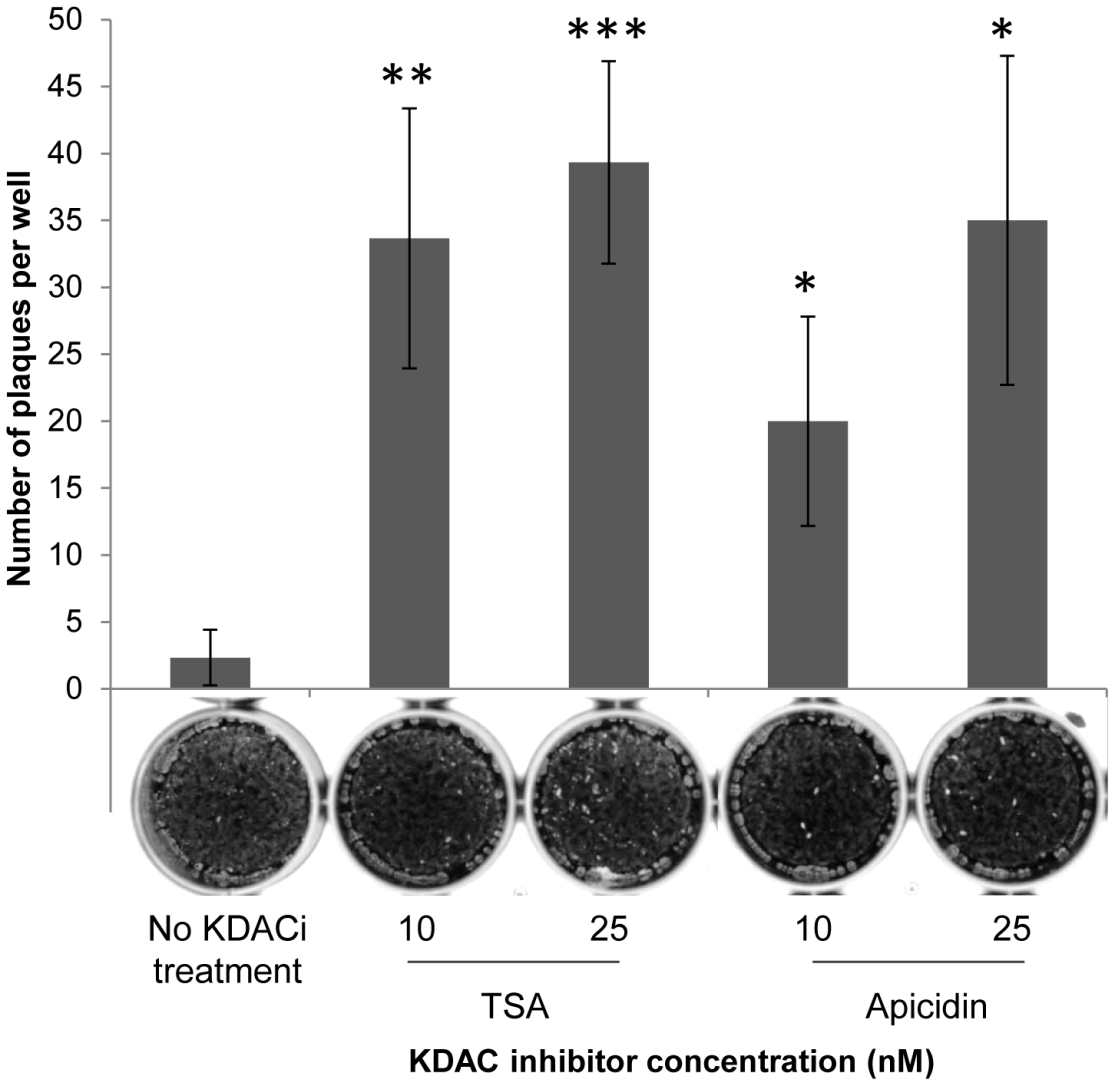
KDAC inhibitors (KDACi) restore replication of dominant-negative GCN5b parasites. _ddHA_GCN5b(E703G) tachyzoites were treated with a range of concentrations of either TSA or apicidin in combination with 500 nM Shield-1. Infected cells with no KDACi were included as a control. The number of parasite plaques in each infected host cell monolayer was counted after 7 days. Representative images of infected wells are depicted underneath the bar for each treatment. Each sample was performed in triplicate, with error bars showing standard deviation. Data shown are from one representative experiment from 3 independent trials that produced similar results. Asterisks denote statistical significance according to two-tailed student t-test; *** p<0.005, **p<0.01, *p<0.05.

We reasoned that if the growth arrest of Shield-treated _ddHA_GCN5b(E703G) parasites was due to hypoacetylation of histones, then inhibition of lysine deacetylases (KDACs) should help restore replication. We therefore incubated _ddHA_GCN5b(E703G) parasites with 500 nM Shield in combination with increasing sublethal concentrations of either apicidin or TSA, two independent, broad-spectrum KDAC inhibitors. Parasite plaque assays were performed one week post-infection. Consistent with results seen in Fig. 1, virtually no plaques were evident in infected monolayers that contained Shield with no KDAC inhibitor (Fig. 4). However, Shield-treated _ddHA_GCN5b(E703G) parasites were able to resume proliferation with the inclusion of either KDAC inhibitor in dose-dependent fashion (Fig. 4).

### Genome-wide localization of GCN5b

To identify genes regulated by GCN5b, we performed a genome-wide ChIP-chip analysis on HA-tagged GCN5b expressing parasites. Immunoprecipitated DNA associated with GCN5b was identified following hybridization to custom Nimblegen microarrays that tile the entire *Toxoplasma* genome. Three replicates were performed. GCN5b was detected at 195 tachyzoite genes in all 3 replicates and 1090 genes in two ChIP-chip replicates, when 0.05 was used as the FDR for significant peaks (Supplemental Dataset S1). Although we observe variability in the location of GCN5b peaks, we detect a statistically significant overlap between the three ChIP-chip replicates that is not present when we randomize the peak positions for statistically significant peaks (FDR<0.05; see methods for details).

**Figure 5:**
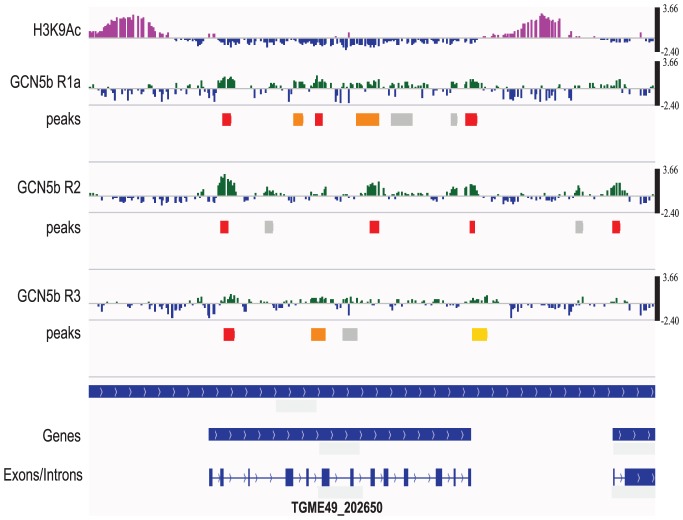
GCN5b associates with genes containing introns, but is not preferentially associated with promoters. The ChIP-chip results for TgME49_202650 are shown. The top tracing shows the H3K9ac ChIP-chip result (purple), which indicates the location of active promoters in *Toxoplasma*. Underneath are the individual results for each of the three ChIP-chip replicates (see Supplemental Data S1) plotted as the log2 ratio between the experimental GCN5b ChIP and input DNA (scale bar on the right). High stringency peaks with FDR<0.05 are shown in red. Peaks with 0.05<FDR<0.1 are shown in orange; 0.1<FDR<0.2 are shown in yellow; and peaks with 0.2<FDR<1 in gray. Only high stringency peaks (FDR<0.05) were used for the statistical analysis performed in this study. The bottom panel displays the gene prediction and the positions of its introns and exons (ToxoDB.org).

It was expected that GCN5b would localize to gene promoters, but GCN5b was also detected in gene bodies, with no detectable preference for gene bodies or intergenic regions (Fig. 5) when significant peaks (FDR<0.05) were statistically compared. Analyses that compared “promoters” (H3K9ac; H3K4me3), “active genes” (H3K4me1), and “centromeres” (CenH3) using genome-wide epigenome mapping yielded similar results [26], [27]. The distance between each GCN5b-associating site (FDR<0.05) and the nearest transcription start site (TSS) was also calculated and plotted as a histogram (Supplemental Figure S3). Results show that enrichment of GCN5b occurs up- and down-stream, rather than at the TSSs. These data are consistent with recent studies showing a role for GCN5 in transcriptional elongation by promoting nucleosome eviction [28]–[30]. Overall, the ChIP-chip results establish that GCN5b is present within or near the loci of tachyzoite genes involved in a wide variety of cellular functions (see Supplemental dataset S1 for breakdown of KEGG and GO classifications of the 195 genes identified in all 3 replicates). Many of the genes are annotated as hypothetical genes of unknown function in the ToxoDB. The high confidence genes coinciding with GCN5b localization are associated with gene expression and RNA processing, as well as metabolic genes, rather than genes linked to virulence.

Although GCN5b did not show a detectable preference for functional regions of the genome, there was a significant association with genes containing introns versus those that do not. By hypergeometric test, each of the three ChIP-chip replicates has a statistically significant (p<0.05) preference for intron-containing genes (5,989) versus those that do not (2,083). While associated with intron-containing genes, GCN5b did not show a statistical association with introns versus exons.

We then performed a more targeted approach to verify if select genes detected by the ChIP-chip study were modulated by GCN5b activity. Primers were designed to amplify selected mRNAs in _ddHA_GCN5b or _ddHA_GCN5b(E703G) parasites in the presence or absence of Shield. Six primer pairs were designed to amplify mRNAs from GCN5b-associated genes and another 6 primer pairs were designed to mRNAs of genes that were not detected in the GCN5b ChIP-chip (Supplemental Table S1). Virtually no changes in mRNA levels were detected in _ddHA_GCN5b parasites regardless of whether GCN5b was detected at the gene locus (Table 1). However, expression levels of mRNAs in _ddHA_GCN5b(E703G) parasites were clearly altered, suggesting a role for GCN5b in gene activation. All 6 genes detected in the GCN5b ChIP-chip had lowered mRNA levels in the _ddHA_GCN5b(E703G) parasites following Shield treatment (Table 1). Genes that were not detected in the GCN5b ChIP-chip experiments generally exhibited no significant difference in mRNA levels in the _ddHA_GCN5b(E703G) parasites following Shield treatment, however one gene showed higher mRNA levels and another showed lower mRNA levels (Table 1). The altered expression of these two genes may be due to sensitivity of the ChIP-chip or indirect effects impacting their mRNA levels. Nevertheless, the results show a general trend that is consistent with the decreased acetylation observed in _ddHA_GCN5b(E703G) parasites leading to decreased transcription of GCN5b-associated genes. This independent qRT-PCR analysis not only highlights the fidelity of the ChIP-chip dataset, but supports the idea that the dysregulation of gene expression induced by the accumulation of _ddHA_GCN5b(E703G) protein contributes to the arrest in parasite replication.

**Table 1:** Levels of select mRNAs detected in _ddHA_GCN5b and _ddHA_GCN5b(E703G) parasites.

Gene ID	Product Description	ddHAGCN5b	ddHAGCN5b
			(E703G)
**Genes detected in GCN5b ChIP-chip**			
TGME49_202370	T-complex protein 1, epsilon subunit	0.9±0.1	**0.4±0.0**
	(TCP-1-epsilon), putative		
TGME49_202360	methylase, putative	1.0±0.1	**0.3±0.1**
TGME49_270840	poly(ADP-ribose) polymerase	1.0±0.2	**0.6±0.0**
	catalytic domain-containing protein		
TGME49_202650*	hypothetical protein	1.0±0.0	**0.3±0.1**
TGME49_253170	zinc carboxypeptidase, putative	1.1±0.1	**0.3±0.1**
**Genes not detected in GCN5b ChIP-chip**			
TGME49_319560	microneme protein MIC3	1.1±0.2	1.3±0.3
TGME49_280500	inorganic anion transporter	0.9±0.2	**0.5±0.3**
TGME49_297060	phosphoglycerate mutase PGMII	1.0±0.3	1.4±0.4
TGME49_289690	glyceraldehyde-3-phosphate	1.0±0.3	0.8±0.3
	dehydrogenase GAPDH1		
TGME49_294200	glucose-6-phosphate	1.2±0.3	1.3±0.5
	1-dehydrogenase		
TGME49_236210	mitochondrial processing peptidase	1.3±0.3	**2.3±0.4**

Values represent fold-change of mRNA levels in parasites treated with Shield for 48 hours relative to vehicle control. The fold change normalized to β-tubulin and the expression level of each gene in EtOH control was set as 1. Student's t-test was performed and p values were <0.05. **Bold** highlights a significant change in mRNA level.

* indicates the gene used in the ChIP analysis shown in Fig. 5.

### GCN5b interacts with a large number of novel proteins, including AP2 factors

As GCN5 KATs lack DNA-binding domains, the complex must be recruited to target genes by virtue of a DNA-bound transcription factor, e.g. GCN4 in *Saccharomyces cerevisiae* or its human counterpart, ATF4 [31]. However, this well-conserved class of transcription factor is not present in Apicomplexa. We therefore performed biochemical purifications of the GCN5b complex from nuclear fractions of intracellular tachyzoites to define the KAT's interactome. Two independent co-immunoprecipitations were performed using RH parasites stably expressing HA-tagged GCN5b. Supplemental dataset S2 is a complete list of proteins identified in each pull down experiment.

As expected, GCN5b itself as well as the known interacting co-activator protein, ADA2-A [20], were identified in each purification. Supporting the idea that DNA-binding proteins would recruit GCN5b to specific gene sites, four AP2 factors were identified in association with GCN5b (AP2IX-7, AP2X-8, AP2XI-2, and AP2XII-4). Another GCN5b-interacting protein with a probable DNA-binding domain is the AT-hook protein (TGME49_109250). Consistent with its frequent localization on gene bodies, GCN5b was associated with RTF1, LEO1, and CTR9, three components of the PAF (polymerase associated factor) complex associated with mRNA elongation [32]. GCN5 activities are typically coordinated with those of SWI/SNF complexes [33], and we detected two distinct SWI/SNF ATPases (TGME49_120300 and TGME49_078440) associated with GCN5b.

**Figure 6:**
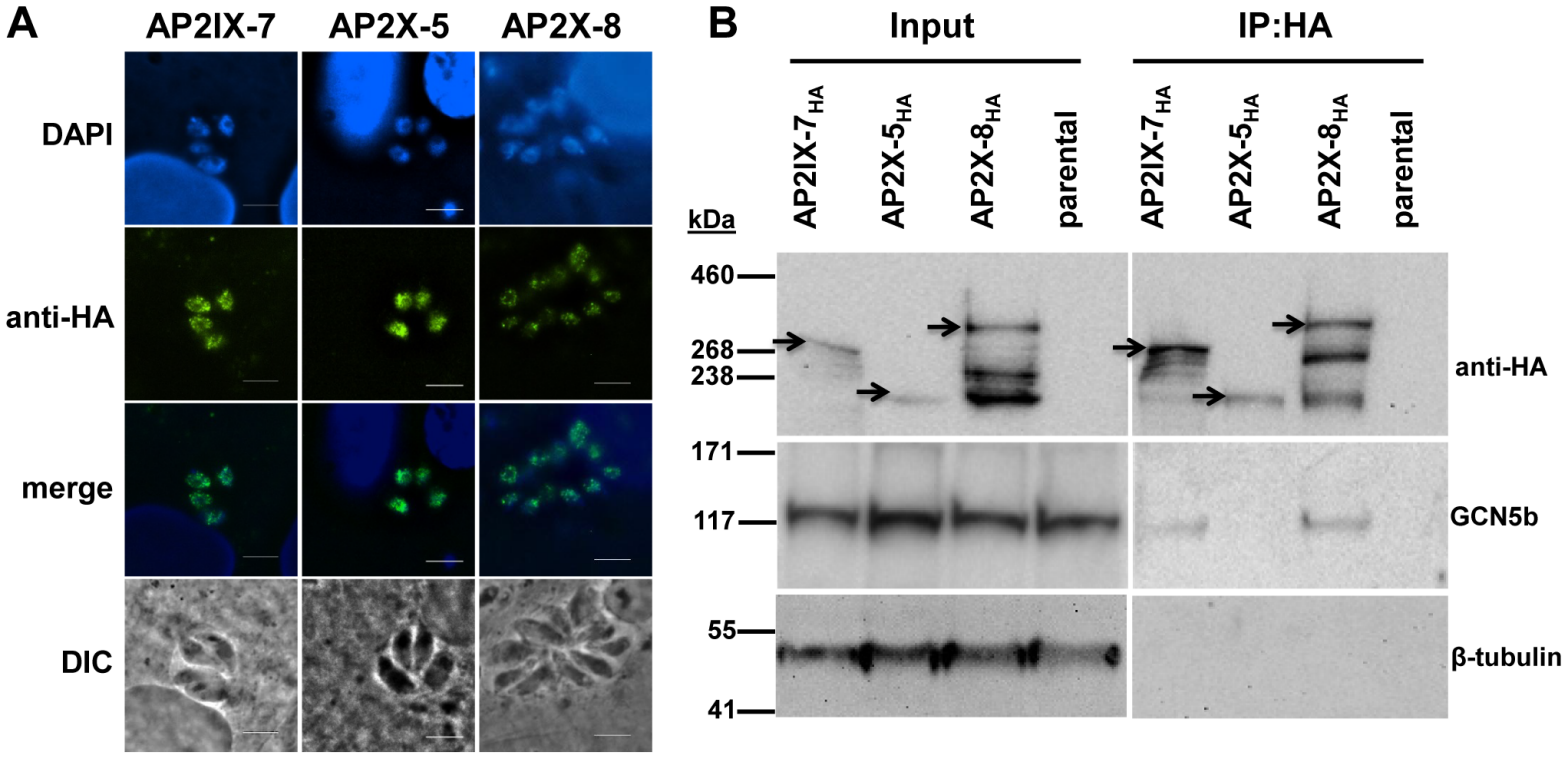
Reciprocal immunoprecipitation confirms the *in vivo* interaction of GCN5b with endogenously HA-tagged AP2IX-7 and AP2X-8. A. IFAs showing localization of each AP2 to the parasite nucleus. Anti-HA (green) shows localization of designate HA-tagged AP2 protein; DAPI (blue) co-stains the nuclei. B. Immunoprecipitations using an anti-HA antibody were performed on parasite lysates made from AP2IX-7_HA_, AP2X-8_HA_, and AP2X-5_HA_ parasites, as well as the parental RHΔ*ku80* line. The immunoprecipitated complexes were analyzed by Western blot using antibodies recognizing anti-HA, GCN5b, or β-tubulin. Arrowheads designate the expected size of each tagged AP2 protein (these AP2 proteins are very large and various breakdown products were observed in the different conditions used to process lysates versus IPs).

The finding that plant-like AP2 factors may partner with KAT complexes to alter transcription is of particular relevance. Associations between GCN5 and AP2 proteins have yet to be demonstrated for any species, including plants. To validate that GCN5b and these AP2 factors reside in the same complex, we endogenously tagged AP2IX-7 and AP2X-8 with a C-terminal 3×HA tag (Fig. 6A). Reciprocal co-immunoprecipitation of each AP2 factor pulled down GCN5b and many of the other proteins seen in the previous GCN5b IPs (Supplemental dataset S2). We refer to proteins that were pulled down consistently in all three purifications as the GCN5b/AP2 “core complex” (Table 2). To further confirm the association of GCN5b with these AP2 factors, we performed Western blots for GCN5b in AP2IX-7_HA_ and AP2X-8_HA_ immunoprecipitates. We also endogenously tagged AP2X-5, an AP2 factor that was not seen in the GCN5b IPs, to serve as a control. As shown in Fig. 6B, GCN5b was detected in a Western blot of HA-immunoprecipitated AP2IX-7_HA_ and AP2X-8_HA_, but not AP2X-5_HA_. Collectively, these results demonstrate specific interactions between GCN5b, AP2IX-7, and AP2X-8. Whether these three proteins interact directly, or through contact with other proteins in the complex, remains to be elucidated.

**Table 2:** GCN5b/AP2 core complex in *Toxoplasma* tachyzoites.

Gene ID	Gene Description
TGME49_017050* #	ADA2-A transcriptional co-activator
TGME49_090630* #	AP2IX-7
TGME49_014960* #	AP2X-8
TGME49_043440* #	GCN5b
TGME49_041850* #	hypothetical
TGME49_080590* #	hypothetical
TGME49_026620* #	hypothetical
TGME49_074180#	hypothetical
TGME49_016750	LEO1 Paf1/RNA polymerase II complex component LEO1
TGME49_078870	MyoF
TGME49_069980	protein transport protein Sec61 alpha subunit isoform 1
TGME49_106060	RON8
TGME49_105850#	RRM protein
TGME49_062620	RRM protein
TGME49_023390#	sec63 domain-containing DEAD/DEAH box helicase
TGME49_030960#	splicing factor 3B subunit 3
TGME49_108890	SPT6 transcription elongation factor SPT6
TGME49_064660	SRS44
TGME49_076180#	TAF1/TAF250
TGME49_118260	TAF5 transcription initiation factor TFIID subunit 5

* denotes a protein also detected in GCN5-B purification performed by Ali Hakimi.

# denotes a protein subject to lysine acetylation.

The GCN5b complex associates with components of the TFIID transcription complex (TAF1/TAF250 and TAF5), affirming its role in facilitating transcriptional activation. Intriguingly, a surprising number of proteins associated with pre-mRNA splicing were also identified in the GCN5b complex (Table 2). In keeping with prior observations that *Toxoplasma* lacks homologues of most proteins found in the GCN5 complexes in other species [20], the majority of proteins in the GCN5b complex are novel interacting partners, with four being hypothetical proteins with unknown function (Table 2).

Twelve of the 20 subunits compromising the GCN5b core complex were detected as acetylated in a previous study (shaded gray in Table 2). Up to four proteins in the GCN5b complex, including GCN5b itself as well as TAF1/TAF250 [34], contain bromodomains that recognize acetylated lysine residues [35] (Supplemental dataset S2). The high degree of acetylated subunits and the presence of multiple bromodomain modules in the complex supports the idea that they participate in intracomplex protein-protein interactions through the binding of acetylated lysines [36].

## Discussion

The objective of this study was to gain a better understanding of the role played by the second GCN5 KAT in *Toxoplasma* parasites through biochemical purification of associating proteins, ChIP-chip analyses, and the generation of mutants. Our findings reveal that GCN5b interacts with a large number of novel proteins and is enriched at genes involved in transcription, translation, and metabolism. Consistent with previous failures to knockout GCN5b, inducible expression of a catalytically dead version acted like a dominant-negative mutant and displayed replicative arrest, supporting that GCN5b is essential in tachyzoites.

The GCN5b interactome is unique and includes components possessing plant-like AP2 DNA-binding domains, thereby providing a probable mechanism by which the complex can be recruited to target promoters. A high-throughput yeast two-hybrid approach previously identified the single *Plasmodium falciparum* GCN5 to be the most interconnected protein in the parasite integrating chromatin modification, transcriptional regulation, mRNA stability, and ubiquitination [23]. The discovery of AP2 factors in the GCN5b interactome prompted us to re-examine the PfGCN5 data, since this analysis was done prior to the identification of AP2 domains. PfGCN5 interacts with two predicted AP2 factors, PF3D7_1007700 and PF3D7_0802100, but they do not have similar DNA-binding domains or other conserved domains that might suggest orthology to the TgAP2s interacting with GCN5b. Interestingly, no other PfGCN5-interacting proteins cross-reference to the GCN5b interacting proteins. This may be due to the different techniques that were used (yeast two-hybrid using only the N-terminal extension of PfGCN5 as bait versus biochemical purification of full-length GCN5b), or could indicate that GCN5 complexes between apicomplexan species have significantly diverged. In support of this, the lengthy N-terminal extensions between PfGCN5 and GCN5b share no obvious sequence homology. Another possibility is that the PfGCN5 complex may be more analogous to that of GCN5a, whose interactome has yet to be resolved.

It is well-established that histone acetylation complexes generally aggregate at gene promoters, but the considerable proportion of GCN5b located within gene bodies is not without precedent. Govind et al. reported that yeast GCN5 plays a role in transcriptional elongation by promoting histone eviction [28]. GCN5 was also found to be predominantly localized to coding regions of highly transcribed genes in fission yeast, where it interplays with an HDAC to modulate H3K14-Ac levels and transcriptional elongation [30]. Interestingly, an Spt6 homologue was purified with the GCN5b complex, a protein that has been implicated in transcription elongation through binding of RNA polymerase II [37]. In other species, GCN5 has also been shown to play a role in co-transcriptional splicing [38]. The disproportionate number of pre-mRNA splicing components that we identified in the GCN5b complex might be suggestive of additional roles for GCN5b in splicing. Although it was not preferentially associated with introns versus exons, GCN5b preferentially associated with genes containing introns in our ChIP-chip analysis, providing further evidence that GCN5b macromolecular complexes may be involved in the modulating splicing. Recently, a novel *Toxoplasma* G1 cell cycle mutant was found to map to an RRM protein that interacts with the splicesome [39]. Further studies are required to delineate the interaction of GCN5b with the splicesome and whether the dominant negative effects of _ddHA_GCN5b(E703G) affect *Toxoplasma* splicing.

It is probable that GCN5b forms multiple complexes and contributes to an assortment of cell biological functions, as seen in other species [40]. The GCN5b-interacting proteins described in this report were isolated from intracellular, replicating tachyzoites. It is possible that GCN5b partners with different components in extracellular tachyzoites or when parasites are subjected to different stress conditions. While our data clearly shows that histone acetylation is decreased in the dominant-negative clone, we cannot conclude that the arrest in parasite replication is solely due to dysregulation of gene expression. Complicating matters is the recent observation that lysine acetylation is widespread on hundreds of non-histone proteins, many of which reside in the parasite nucleus [36]. It is conceivable that GCN5b has non-histone substrates and decreased efficiency in acetylation of those substrates contributes to the replication arrest in the dominant-negative parasites.

*Toxoplasma* is unique as a lower eukaryote to possess a pair of GCN5 KATs. Studies to date suggest that these two GCN5 KATs have non-redundant functions in tachyzoites. GCN5b was not sufficient to compensate for a lack of GCN5a, which is required for adequate responses to alkaline stress [5]. Similarly, GCN5a is not able to compensate when the function of GCN5b is attenuated through expression of a dominant-negative version. It has been previously reported that inhibition of parasite histone modifying enzymes is deleterious to protozoan pathogens [41]. Our findings suggest that pharmacological inhibition of GCN5b or disruption of the GCN5b complex may be novel avenues for therapy against toxoplasmosis.

## Materials and Methods

### Parasite culture and methods

All *Toxoplasma* lines (RH strain) were propagated in monolayers of human foreskin fibroblasts (HFFs) in Dulbecco modified Eagle's medium (DMEM) supplemented with 1% heat-inactivated fetal bovine serum (Gibco/Invitrogen). Cultures were maintained in a humidified, 37°C incubator with 5% CO_2_. To isolate parasites for experiments, intracellular tachyzoites were harvested through syringe passage of infected host cells followed by filtration through a 3 micron filter [42]. Where designated, Shield-1 (CheminPharma), dissolved in ethanol, was added to culture medium. For some experiments, KDAC inhibitors were added to the culture: TSA (Sigma #T8552) or apicidin (Calbiochem #178276). Plasmids were introduced into *Toxoplasma* via electroporation, subjected to drug selection (20 µM chloramphenicol or 1 µM pyrimethamine), and cloned by limiting dilution as previously described [42]. Parasite replication assays were performed as previously described [5], [43].

### Generation of transgenic parasite lines

#### Ectopic expression of inducible forms of GCN5b

We constructed vectors that would express recombinant versions of GCN5b fused to a destabilization domain (dd) and HA epitope tag at the N-terminal end (ddHA). Total RNA was purified from parasites using RNeasy® Plus Mini Kit (Qiagen) then made into cDNA with random primers using Omniscript® RT Kit (Qiagen). The GCN5b coding sequence was amplified from cDNA, engineering an HA epitope (italics) and NsiI restriction site (underscored) at the 5′ end and an AvrII restriction site at the 3′ end (sense primer: 5′-ATGCAT *TACCCGTACGACGTCCCGGACTACGCG*GCGCCTTCAGAGTGTCCCAGCGACGCG; anti-sense primer: 5′-CCTAGGCTAGAAAAATGTCGGATGCTTCGCGCCCACAAGCCCCTCGTCTCC). The dd fragment was amplified from plasmid pLIC.2×HA-DD::DHFR (kindly provided by Dr. Michael White [25]), engineering an NdeI restriction site (underscored) at the 5′ end and NsiI and AvrII restriction sites (underscored) at the 3′ end (sense primer: 5′-CATATGAAAATGGCGGGAGTGCAGGTGGAAACCATCTCC; anti-sense primer: 3′-CCTAGGATCGATATGCATTTCCGGTTTTAGAAGCTCCACATCGAAGACGAGAGTGGC). The amplified dd fragment was cloned into a *Toxoplasma* ptubXFLAG::CAT expression vector, which contains a tubulin promoter and CAT minigene for chloramphenicol selection, using the NdeI and AvrII restriction sites [21], followed by insertion of the HA-GCN5b coding sequence at the NsiI and AvrII sites. The QuikChange™ Site-Directed Mutagenesis Kit (Agilent Technologies) was used to create a point mutation (E703G) in the GCN5b coding region in this construct (sense primer: 5′-CAGCAGAAATTCGCCGGCATCGCTTTCCTCGCG; anti-sense primer: 5′-CGCGAGGAAAGCGATGCCGGCGAATTTCTGCTG). Plasmids were linearized using NotI prior to transfection into parasites by electroporation.

#### Endogenous tagging of AP2 factors

Genomic DNA from the 3′ end of AP2X-8 (TGGT1_125480), AP2IX-7 (TGGT1_032510), or AP2X-5 (TGGT 1_068820) was amplified from parental strain RH**Δ** *ku80* and cloned into the pLIC.HA3.DHFR vector (kindly provided by Michael White) at the PacI site using methods previously described [44]. Primers for the genomic fragments were as follows. AP2IX-7 (sense primer 5′-TACTTCCAATCCAATTTAATGCCGGCGGCATGAGCTTCAGTT; antisense 5′-TCCTCCACTTCCAATTTTAGCAAAGTCTTCGTCAACAACGAACTTGC). AP2X-5 (sense primer 5′-TACTTCCAATCCAATTTAATGCAGCTACACGACAGCGACGGA; antisense 5′-TCCTCCACTTCCAATTTTAGCGGGCCAAAACGAGGAAGCGAG). AP2X-8 (sense 5′-TACTTCCAATCCAATTTAATGCAGCGCAGAAGCTGCAGAACC; antisense 5′-TCCTCCACTTCCAATTTTAGCTCCCCCCGCGCCTCTCAC). Fifty µg plasmid DNA of AP2IX-7_HA_, AP2X-5_HA_, and AP2X-8_HA_ were linearized by *Nco*I, *Bpu10*I, or *Nsi*I, respectively, and then electroporated into RH**Δ** *ku80* parasites. Following three passages under 1.0 µM pyrimethamine selection, parasites were cloned by limiting dilution.

### Immunofluorescence assays

Immunofluorescence assays (IFA) were performed as previously described [21]. Briefly, HFF monolayers grown on coverslips were inoculated with the designated parasite line, sometimes containing Shield-1 or EtOH vehicle. After removal of culture medium, infected HFFs were fixed in 3% paraformaldehyde for 10 min and then were permeabilized with 0.3% Triton X-100 for 10 min. For visualization of HA-fusion proteins, rat monoclonal anti-HA primary antibody (Roche #11867423001) was applied at 1∶2,000 followed by goat anti-rat Alexa Fluor 488 secondary antibody at 1∶2,000 (Invitrogen #A-11006). Nuclei were co-stained with 4′,6-diamidino-2-phenylindole (DAPI). Samples were visualized using a Leica DMLB fluorescent microscope.

### Western blotting

Western blots to monitor Shield-based protein stabilization were performed by resolving 50 µg parasite lysate on a 4–12% Tris-acetate polyacrylamide gradient gel (Invitrogen) and probing with 1∶2,000 rat anti-HA monoclonal antibody as the primary antibody (Roche #11867423001). Analysis of histones and tubulin used the following primary antibodies: rabbit polyclonal anti-H3 antibody (Abcam #ab1791, 1∶2,000), rabbit polyclonal antibodies against acetyl H3K9 (Millipore #06-942, 1∶2,000), acetyl H3K14 (Millipore #06-911, 1∶2,000), acetyl H3K18 (Abcam #ab1191, 1∶2,000), and rabbit polyclonal antibody against *Toxoplasma* β-tubulin (kindly provided by Dr. David Sibley, 1∶5,000). Anti-rat or anti-rabbit antibodies conjugated with horseradish peroxidase (GE Healthcare) were used as secondary antibodies at 1∶5,000. The blots were visualized using Chemiluminescence Western Blot Substrate (Pierce).

### Purification of GCN5b and AP2 complexes

The GCN5b complex was purified from RHΔ*hxgprt* parasites stably transfected to ectopically express an Nt HAmyc-tagged form of full-length GCN5b driven by the *Toxoplasma* tubulin (*TUB1*) promoter (described in [21]). Two experiments were performed following an initial co-IP done by Dr. Ali Hakimi. AP2 factor complexes were independently purified from parasites expressing AP2X-8 and AP2IX-7 endogenously tagged with HA (see above). Parental RHΔ*hxgprt* (for GCN5b line) and RHΔ*ku80* (for AP2 lines) were run in parallel as negative controls. Large-scale tachyzoite cultures were grown in monolayers of HFF cells at 37°C for 42 hours post-infection. Prior to egress, culture medium was removed and the cell monolayers were washed once with PBS, scraped into cold PBS and then collected by centrifugation at 4°C for 10 min at 700×*g*. The cell pellets were resuspended in 25 ml cold PBS, and sequentially passed through 20/23/25-gauge needles in a 30 ml syringe to release intracellular parasites from the host cells. To prepare parasite nuclear extracts, 3×10^9^ parasites were incubated 5 min on ice in lysis buffer A (0.1% [v/v] NP-40, 10 mM HEPES pH 7.4, 10 mM KCl, 10% [v/v] glycerol, 20 mM sodium butyrate, plus protease inhibitors), and the nuclei were pelleted by centrifugation 6,000×*g* for 8 min at 4°C. The parasite nuclei were then incubated 30 min at 4°C in lysis buffer B (0.1% [v/v] NP-40, 10 mM HEPES pH 7.4, 400 mM KCl, 10% [v/v] glycerol, 20 mM sodium butyrate, plus protease inhibitors) with rotation, and subjected to five freeze-thaw cycles followed by vortexing for 1 min at 4°C before freezing. The nuclear extracts were clarified by centrifugation at 12,000×*g* for 30 min at 4°C. The mixture of the clarified nuclear extracts (1 part) with lysis buffer A (2 parts) was used for co-immunoprecipitation. Nuclear extracts were incubated with mouse monoclonal anti-HA-tag magnetic beads (μMACS Anti-HA Microbeads; Miltenyi Biotec) overnight at 4°C with rotation. After the beads were washed 4 times with cold wash buffer 1 (150 mM NaCl, 1% NP-40, 0.5% sodium deoxycholate, 0.1% SDS, 50 mM Tris-HCl pH 8.0) and once with wash buffer 2 (20 mM Tris-HCl pH 7.5) by using μ Column (pre-washed with buffer containing 0.1% [v/v] NP-40, 10 mM HEPES pH 7.4, 150 mM KCl, plus protease inhibitors) in the magnetic field of the separator, the bound proteins were eluted from the magnetic beads by applying 50 µl of Laemmli's sample buffer (pre-heated to 95°C) to the column. Eluted proteins were separated by SDS-PAGE (Any kD™ precast polyacrylamide gel; Bio-Rad), and stained with Coomassie blue (GelCode Blue Stain Reagent; Pierce). The entire length of each sample lane was systematically cut into 24 slices and the gel slices were maintained in MilliQ water until trypsin digestion.

### Liquid chromatography coupled to mass spectrometry (LC/MS-MS)

Proteins from a Coomassie-stained gel were reduced then alkylated with TCEP and iodoacetamide prior to digestion with trypsin. Trypsin (Sequencing grade, Promega) digestion was carried out for 1 hour at 50°C using 10 ng/ul solution in 25 mM ammonium bicarbonate/0.1%ProteaseMax (Promega). The resulting digest was then diluted with 2%Acetonitrile/2%TFA prior to LC-MS/MS analysis. Nanospray LC-MS/MS was performed on a LTQ linear ion trap mass spectrometer (LTQ, Thermo, San Jose, CA) interfaced with a Rapid Separation LC3000 system (Dionex Corporation, Sunnyvale, CA). Thirty-five µL of the sample was loaded on an Acclaim PepMap C18 Nanotrap column (5 µm, 100 Å,/100 µm i.d. ×2 cm) from the autosampler with a 50 µl sample loop with the loading buffer (2% Acetonitrile/water+0.1% trifluoroacetic acid) at a flow rate of 8 µl/min. After 15 minutes, the trap column was switched in line with the Acclaim PepMap RSLC C18 column (2 µm, 100 Å, 75 µm i.d. ×25 cm) (Dionex Corp). The peptides are eluted with gradient separation using mobile phase A (2% Acetonitrile/water +0.1% formic acid) and mobile phase B (80% acetonitrile/water+0.1% formic acid). Solvent B was increased from 2 to 35% over 40 min, increased to 90% over a 5-min period and held at 90% for 10 min at a flow rate of 300 nL/min. The 10 most intense ions with charge state from +2 to +4 determined from an initial survey scan from 300–1600 m/z, were selected for fragmentation (MS/MS). MS/MS was performed using an isolation width of 2 m/z; normalized collision energy of 35%; activation time of 30 ms and a minimum signal intensity of 10,000 counts. The dynamic exclusion option is enabled. Once a certain ion is selected once for MS/MS in 7 sec, this ion is excluded from being selected again for a period of 15 sec.

Mgf files were created from the raw LTQ mass spectrometer LC-MS/MS data using Proteome Discoverer 1.2 (ThermoScientific). The created mgf files were used to search the Toxo_Human Combined database [45] using the in-house Mascot Protein Search engine (Matrix Science) with the following parameters: trypsin 2 missed cleavages; fixed modification of carbamidomethylation (Cys); variable modifications of deamidation (Asn and Gln), pyro-glu (Glu and Gln) and oxidation (Met); monoisotopic masses; peptide mass tolerance of 2 Da; product ion mass tolerance of 0.6 Da. The final list of identified proteins was generated by Scaffold 3.5.1 (Proteome Software) with following filters: 99% minimum protein probability, minimum number peptides of 2 and 95% peptide probability. All searches were performed against a decoy database and yielded no hits (i.e. a FDR of 0%). For final presentation of data, proteins appearing in the negative control parental lines and human proteins were treated as non-specific contaminants.

### Immunoprecipitation of GCN5b from parasites containing HA-tagged AP2 factors

Parasites from AP2IX-7_HA_, AP2X-5_HA_, AP2X-8_HA_, and parental RHΔ*ku80* lines were harvested in lysis buffer (150 mM NaCl, 50 mM TrisCl pH 7.4, 0.1% NP-40) with 1× protein inhibitor cocktail (Sigma) and 1 mM PMSF. The lysates were then sonicated and centrifuged to remove the insoluble fraction. Immunoprecipitations were performed using anti-HA high affinity matrix (Roche) and 300 µg total parasite protein. After overnight incubation at 4°C, the beads were washed 3× in lysis buffer and treated at 95°C for 10 minutes to elute proteins. Eluted proteins were resolved by SDS-PAGE and analyzed by Western blot with HA (1∶2,000), GCN5b (1∶500), or β-tubulin (1∶1,000) antibodies.

### Chromatin immunoprecipitation and chip hybridization (ChIP-chip)

ChIP was performed as described [26] with some modifications. Briefly, intracellular tachyzoites grown in HFF cell monolayers for 42 hours were cross-linked for 10 min with 1% formaldehyde in PBS and quenched with 125 mM glycine for 5 min at room temperature. The cell monolayers were washed with PBS, scraped into PBS and then collected by centrifugation at 4°C for 10 min at 700×*g*. The cell pellets were resuspended in cold PBS, and sequentially passed through 20/23/25-gauge needles in a syringe to release intracellular parasites from the host cells. The parasites were then centrifuged at 4°C for 15 min at 700×*g*, resuspended in lysis buffer (50 mM HEPES, pH 7.5, 150 mM NaCl, 1% NP-40, 0.1% SDS, 0.1% sodium deoxycholate, 1 mM EDTA, plus protease inhibitors), and the chromatin was sheared by sonication yielding DNA fragments of 500–1,000 bp. The chromatin was clarified by centrifugation at 12,000×*g* for 10 min at 4°C; 10% of the clarified chromatin was saved as the input sample and the remaining 90% was used for immunoprecipitation.

Immunoprecipitations were performed with mouse monoclonal anti-HA-tag magnetic beads (μMACS Anti-HA Microbeads; Miltenyi Biotec) overnight at 4°C with rotation, washed extensively, and the GCN5b chromatin was eluted with 1% SDS in TE buffer. Both input and GCN5b chromatin were reverse cross-linked by incubation overnight at 65°C and purified using the Qiagen MinElute PCR purification kit. Purified DNA was amplified by using GenomePlex Complete Whole Genome Amplification kit (WGA2; Sigma) and amplified DNA was further purified using the Qiagen MinElute PCR purification kit.

The genome-wide chip was designed using the Nimblegen isothermal protocol design based upon Release 4.1 of the *Toxoplasma* ME49 genome with a total of 732,672 toxo-specific probes with average spacing of probes every 86 bp using an estimated genome size of 63 Mb. Chip design is available under GEO identifier GPL15563 and GPL15564 and the data is supplied in supplemental dataset S1. H3K4me3, H3K9ac, H3K4me1, H3K9me2, and CenH3 data are also accessible at www.toxodb.org. Hybridization to arrays was performed using standard Nimblegen protocols at Nimblegen or in the Albert Einstein College of Medicine Epigenomics Facility as described in [26] with an input DNA and experimental DNA hybridization performed simultaneously for each experiment on the same chip. ChIP-chip analysis was performed three times. Microarrays were scanned once with excitation at 635 nm for the immunoprecipitated DNA and 532 nm for total genomic DNA. The ratio of probe intensities is calculated and then log-base 2 transformed. After this, the bi-weight mean is calculated for all of the probe ratios. This mean is then subtracted from each of the probe ratios in order to scale the data.

GCN5b localization within the genome was determined using NimbleScan's peak calling algorithm. The algorithm employs a sliding window to look for consecutive probes with high ratios. The threshold for high ratio is percentage of the theoretical maximum ratio value (mean plus six standard deviations). The threshold steps down from 90% to 15% to calculate the significance of the peak. The false discovery rate (FDR) was estimated by randomly permuting probe ratio values. Positions of peaks with a false-discovery rate (FDR)<0.05 within each chromosome were compared between biological replicates using custom Perl scripts.

Reproducibility of GCN5b peaks was judged using the “makeVennDiagram” function from the ChIPpeakAnno package of the Bioconductor project [46]. We first selected those peaks that have an FDR below 0.05. We then identified peaks from different replicates that overlap by 50 nucleotides or more. Finally, we used the hypergeometric distribution to calculate the significance of the overlap. The probability of finding the number of overlapping peaks by chance was calculated as: 1 vs 2 7.33 E-105; 2 vs 3 2.56 E-19; 1 vs 3 0.0005. Association between GCN5b and specific genes was established by using the “findOverlappingPeaks” function from the ChIPpeakAnno package of the Bioconductor project. We used the gene annotations from ToxoDB, release 6.1, ME49 strain. Gene associations for each replicate were determined independently.

We used two different randomization strategies to test the reproducibility of our GCN5b ChIP. First, we reassigned the starting position of each of our significant peaks (FDR< = 0.05) to a random probe from the microarray while keeping the peak widths the same. We used the hypergeometric distribution to calculate the significance of the overlap with the randomly selected peaks in each replicate. The probability of chance overlap in each of these cases was close to one. In a second test, we randomly selected an equal number of peaks from each replicate, regardless of FDR. These peaks were reanalyzed using the hypergeometric distribution to calculate the significance of the overlap with the peaks in each replicate. These randomizations also resulted in a probability of close to one that the overlap was due to chance. Both randomization tests were performed 1,000 times.

For examination of GCN5b peaks with introns or genes with introns, gene models and annotations from www.toxodb.org V6 were used. Significance of overlaps was determined by hypergeometric test for intron-containing genes (5,989) versus intronless genes (2,083). To further test this, we selected random peaks from each experiment rather than only the significant peaks (FDR<0.5%). We repeated the same test with the randomized peaks. Using the same methods, we also tested for an association between GCN5b binding with introns or exons. We also tested for an association with active promoters (as defined by dual marking with H3K9ac and H3K4me3 [26]), active coding regions (defined by the H3K4me1 mark [26]), or centromeres [27] and measured the distribution of GCN5b peaks from the inferred transcription start site using data from Yamagishi *et al.* [47].

### Quantitative reverse-transcriptase PCR (qRT-PCR)

1.0 µg of total RNA purified from intracellular parasites was transcribed into cDNA using Omniscript reverse transcriptase with oligo-dT primers according to the manufacturer's protocol (Qiagen). qRT-PCR was performed in 25 µl volume reactions containing SYBR Green PCR Master Mix (Applied Biosystems), 0.5 mM of each forward and reverse primer (Supplemental Table S1), and 1.0 µl of a 1∶10 dilution of cDNA. Target genes were amplified using the 7500 Real-time PCR system and analyzed with relative quantification software (7500 software v2.0.1, Applied Biosystems). The ratio of mRNA levels in Shield-treated parasites versus EtOH-treated parasites was calculated using *Toxoplasma* β-tubulin as an internal control for normalization (GCN5b was not detected at β–tubulin in any of the three ChIP-chip experiments). Reactions were performed in triplicate and Student's t-test was applied to RT-PCR data.

## Supporting Information

Dataset S1 ChIP-chip of GCN5b. Three replicates of GCN5b chromatin immunoprecipitations were hybridized to a genome-wide chip. Genes identified in each of the replicates are indicated by an X for each replicate. Version 6.1 *Toxoplasma* identifications were used. GO terms as provided at ToxoDB.org are indicated. KEGG pathways and GO terms are summarized for the 195 genes identified by all 3 replicates of ChIP-chip.
(XLSX)

Dataset S2 Proteomics of GCN5b, AP2IX-7, and AP2X-8 identifies a core macromolecular complex. Two replicates of GCN5b pull-downs were performed. Proteins detected in one (1/2) or both (2/2) of the GCN5b IPs are indicated as such. Proteins that were also identified in either of the AP2 pull-downs are indicated by an X. All proteins were identified by at least 2 independent peptides that were assigned with 95% confidence using the Mascot search of the EPIC-DB custom *Toxoplasma* database [45]. An asterisk denotes a protein that was also detected in independent GCN5b purification performed by Ali Hakimi. For additional details of data, please consult http://toro.aecom.yu.edu/cgi-bin/biodefense/main.cgi.
(XLSX)

Figure S1 *In vitro* HAT assays using recombinant histone H3 and either purified _ddHA_GCN5b or _ddHA_GCN5b(E703G). Proteins were purified from parasites cultured in the presence of 500 nM Shield for 48 hours. KAT reactions were analyzed by Western blotting with antibody recognizing acetylated H3. Parental strain was used as a negative control. Anti-HA was used to show that the same approximate amount of protein was used in each assay.
(PDF)

Figure S2 Stalled _ddHA_GCN5b(E703G) parasites resume replication if Shield is removed. Infected monolayers were treated with 500 nM Shield, vehicle, or 1 µM pyrimethamine (pyr). After 48 hours, media was exchanged that lacked Shield or contained 500 nM Shield. Parasite plaques were counted two days later. Graph shows results of a representative experiment performed in triplicate. A representative infected monolayer is shown below the graph.
(PDF)

Figure S3 GCN5b distribution around transcription start sites (TSS). The distance between each GCN5b-associating site (FDR<0.05) and the nearest TSS (based on data in Yamagishi et al. 2010) was calculated and plotted as a histogram.
(PDF)

Table S1 List of primers used in qRT-PCRs to evaluate ChIP-chip results.
(DOCX)
