# Supplementary material for: Lysine Acetyltransferase GCN5b Interacts with AP2 Factors and Is Required for Toxoplasma gondii Proliferation
Source: PLoS Pathog. 2014 Jan 2;10(1):e1003830. doi: 10.1371/journal.ppat.1003830 (PMC3879359; doi:10.1371/journal.ppat.1003830)
Supplement: Figure S1 — In vitro HAT assays using recombinant histone H3 and either purified ddHAGCN5b or ddHAGCN5b(E703G). Proteins were purified from parasites cultured in the presence of 500 nM Shield for 48 hours. KAT reactions were analyzed by Western blotting with antibody recognizing acetylated H3. Parental strain was used as a negative control. Anti-HA was used to show that the same approximate amount of protein was used in each assay. (PDF) [file ppat.1003830.s003.pdf]

Supplemental Figure S1

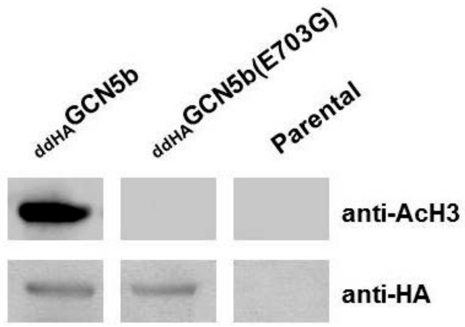

Figure S1. *In vitro* HAT assays using recombinant histone H3 and either purified $_{ddHA}$GCN5b or $_{ddHA}$GCN5b(E703G). Proteins were purified from parasites cultured in the presence of 500 nM Shield for 48 hours. KAT reactions were analyzed by Western blotting with antibody recognizing acetylated H3. Parental strain was used as a negative control. Anti-HA was used to show that the same approximate amount of protein was used in each assay.
