# Supplementary material for: Lysine Acetyltransferase GCN5b Interacts with AP2 Factors and Is Required for Toxoplasma gondii Proliferation
Source: PLoS Pathog. 2014 Jan 2;10(1):e1003830. doi: 10.1371/journal.ppat.1003830 (PMC3879359; doi:10.1371/journal.ppat.1003830)
Supplement: Figure S2 — Stalled ddHAGCN5b(E703G) parasites resume replication if Shield is removed. Infected monolayers were treated with 500 nM Shield, vehicle, or 1 µM pyrimethamine (pyr). After 48 hours, media was exchanged that lacked Shield or contained 500 nM Shield. Parasite plaques were counted two days later. Graph shows results of a representative experiment performed in triplicate. A representative infected monolayer is shown below the graph. (PDF) [file ppat.1003830.s004.pdf]

Supplemental Figure S2

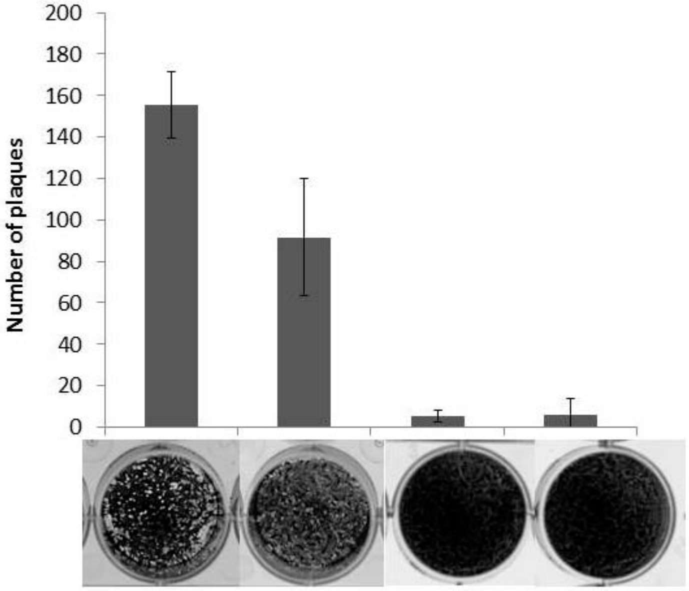

1st 48hrs	- Shield	+ Shield	+ Shield	+ pyr
2nd 48hrs	- Shield	- Shield	+ Shield	- pyr

Figure S2. Stalled $_{ddHA}$GCN5b(E703G) parasites resume replication if Shield is removed. Infected monolayers were treated with 500 nM Shield, vehicle, or 1 µM pyrimethamine (pyr). After 48 hours, media was exchanged that lacked Shield or contained 500 nM Shield. Parasite plaques were counted two days later. Graph shows results of a representative experiment performed in triplicate. A representative infected monolayer is shown below the graph.
