# Supplementary material for: Lysine Acetyltransferase GCN5b Interacts with AP2 Factors and Is Required for Toxoplasma gondii Proliferation
Source: PLoS Pathog. 2014 Jan 2;10(1):e1003830. doi: 10.1371/journal.ppat.1003830 (PMC3879359; doi:10.1371/journal.ppat.1003830)
Supplement: Figure S3 — GCN5b distribution around transcription start sites (TSS). The distance between each GCN5b-associating site (FDR<0.05) and the nearest TSS (based on data in Yamagishi et al. 2010) was calculated and plotted as a histogram. (PDF) [file ppat.1003830.s005.pdf]

Supplemental Figure S3

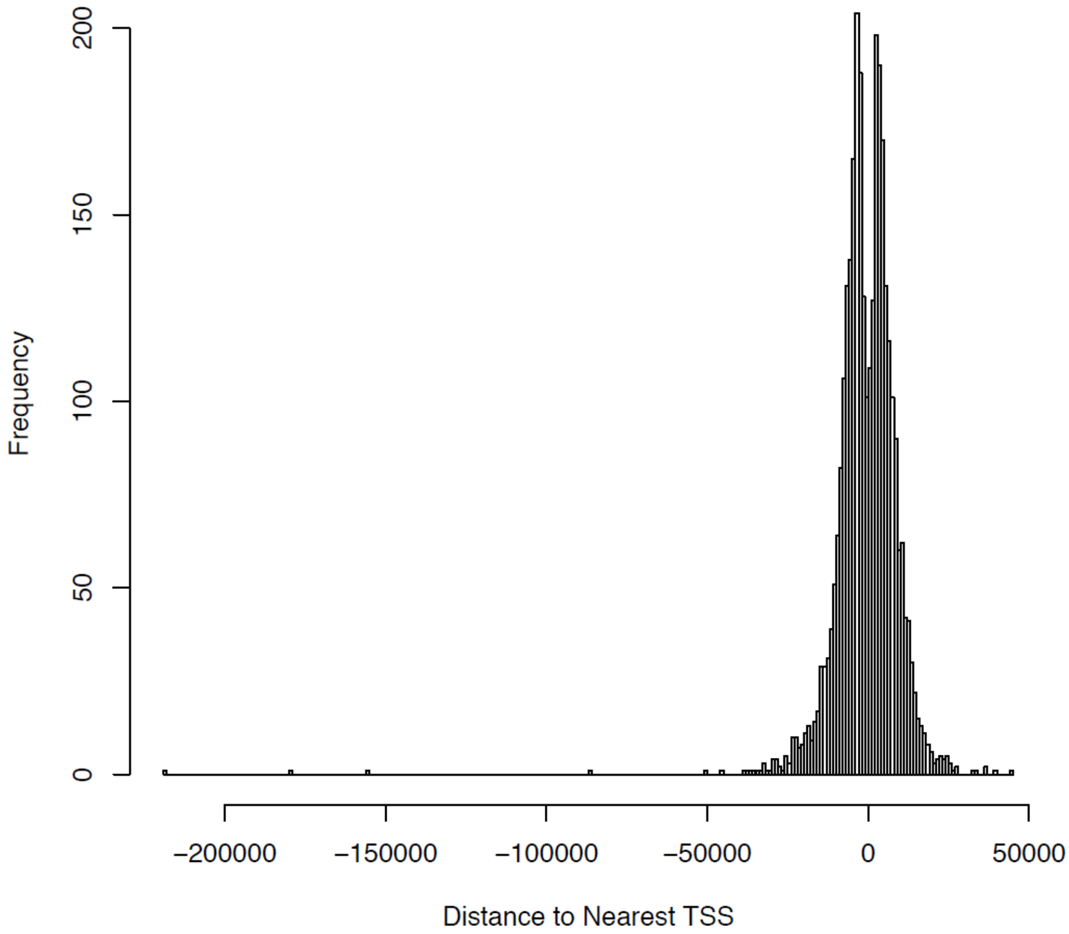

Figure S3. GCN5b distribution around transcription start sites (TSS). The distance between each GCN5b-associating site (FDR < 0.05) and the nearest TSS (based on data in Yamagishi et al. 2010) was calculated and plotted as a histogram.
