# Supplementary material for: Lysine Acetyltransferase GCN5b Interacts with AP2 Factors and Is Required for Toxoplasma gondii Proliferation
Source: PLoS Pathog. 2014 Jan 2;10(1):e1003830. doi: 10.1371/journal.ppat.1003830 (PMC3879359; doi:10.1371/journal.ppat.1003830)
Supplement: Table S1 — List of primers used in qRT-PCRs to evaluate ChIP-chip results. (DOCX) [file ppat.1003830.s006.docx]

**Table S1. List of primers used in qRT-PCRs**

| Primer sequence(5'-3') | Primer name |  |
| --- | --- | --- |
| GAACGGCGTCGTGCTACAC | TGME49_202370F |  |
| TTCGTCACCGATGGCATTT | TGME49_202370R |  |
| TTGGCACAGCGCATCAGTT | TGME49_294550F |  |
| TCGATCCACTCTGCCAAAGTC | TGME49_294550R |  |
| GGAGACACAAAAGACGCAAAGC | TGME49_202360F |  |
| CCCTACGCCGCATCCA | TGME49_202360R |  |
| CTCTTGGCAAGCCGTACGTT | TGME49_270840F |  |
| CCCCACACCGAACAGACTCT | TGME49_270840R |  |
| TTTGACTCGGCGAAATCGA | TGME49_202650F |  |
| GGTGCCAGTGACGATCAAGA | TGME49_202650R |  |
| CCCCGCCAGGCAAGAG | TGME49_253170F |  |
| GAAATGGAGGAGACAGAGCAGAA | TGME49_253170R |  |
| GCATCAGCGCGTCATCCT | TGME49_319560R |  |
| CTCGGATCCCGATTCTCCTT | TGME49_319560R |  |
| GTCGGAGCAGCCAACACAGT | TGME49_280500F |  |
| CCCCCCACCTTTAGATTCAAG | TGME49_280500R |  |
| GGACGAGGCGGAATTGAAG | TGME49_297060F |  |
| TGTGCATGCGGCTTTCTTAT | TGME49_297060R |  |
| TTTTGCTTGGGATTCGAGGAT | TGME49_289690F |  |
| TGCAGGGTAACGATCAAAAAATG | TGME49_289690R |  |
| TCGACGAAGAGACAGGAAATGA | TGME49_294200F |  |
| CATGCGCTCCTGGATAAACA | TGME49_294200R |  |
| ACCTGTCGTGTGGTGTTTCTTCT | TGME49_236210F |  |
| GAAACCACACGCGAAACTGA | TGME49_236210R |  |
| ATGTTCCGTGGTCGCATGT | TUB FWD |  |
| TGGGAATCCACTGAACGAAGT | TUB REV |  |
